# Exploratory-Phase-Free Estimation of GP Hyperparameters in Sequential Design Methods—At the Example of Bayesian Inverse Problems

**DOI:** 10.3389/frai.2020.00052

**Published:** 2020-08-13

**Authors:** Michael Sinsbeck, Marvin Höge, Wolfgang Nowak

**Affiliations:** Department of Stochastic Simulation and Safety Research for Hydrosystems (LS^3^), Institute for Modeling Hydraulic and Environmental Systems, University of Stuttgart, Stuttgart, Germany

**Keywords:** Gaussian process emulators, sequential design of computer experiments, adaptive sampling, hyperparameter estimation, Bayesian inference

## Abstract

Methods for sequential design of computer experiments typically consist of two phases. In the first phase, the exploratory phase, a space-filling initial design is used to estimate hyperparameters of a Gaussian process emulator (GPE) and to provide some initial global exploration of the model function. In the second phase, more design points are added one by one to improve the GPE and to solve the actual problem at hand (e.g., Bayesian optimization, estimation of failure probabilities, solving Bayesian inverse problems). In this article, we investigate whether hyperparameters can be estimated without a separate exploratory phase. Such an approach will leave hyperparameters uncertain in the first iterations, so the acquisition function (which tells where to evaluate the model function next) and the GPE-based estimator need to be adapted to non-Gaussian random fields. Numerical experiments are performed exemplarily on a sequential method for solving Bayesian inverse problems. These experiments show that hyperparameters can indeed be estimated without an exploratory phase and the resulting method works almost as efficient as if the hyperparameters had been known beforehand. This means that the estimation of hyperparameters should not be the reason for including an exploratory phase. Furthermore, we show numerical examples, where these results allow us to eliminate the exploratory phase to make the sequential design method both faster (requiring fewer model evaluations) and easier to use (requiring fewer choices by the user).

## 1. Introduction

Methods for Sequential Design of Experiments (SDoE) exist for a variety of problems, such as optimization (called *Bayesian optimization*) (Kushner, 1964; Jones et al., 1998; Williams et al., 2000; Mockus, 2012; Shahriari et al., 2016; Frazier, 2018), contour estimation (Ranjan et al., 2008; Picheny et al., 2010), estimation of failure probabilities (Bichon et al., 2008; Bect et al., 2012; Balesdent et al., 2013), value of information analysis (Myklebust et al., 2020), and Bayesian inverse problems (Sinsbeck and Nowak, 2017; Damblin et al., 2018; Teckentrup, 2019). In all of these methods, the expensive-to-evaluate model function is described by a Gaussian process emulator (GPE). Alternative to using a GPE, other emulators like neural networks were successfully applied to the same kind of tasks (e.g., Snoek et al., 2015). Here, for SDoE, we focus on the GPE as typical stochastic emulator (Sinsbeck and Nowak, 2017) to sequentially determine the design points for a series of model evaluations. The GPE itself can be thought of as an input to the sequential design method.

**Figure 1:**
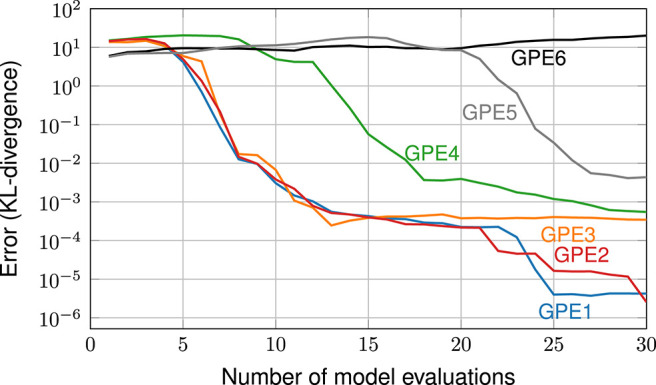
The performance of a sequential design of experiment is highly sensitive to the choice of the GPE. Copyright © 2017 Society for Industrial and Applied Mathematics and American Statistical Association. Reprinted with permission from Sinsbeck and Nowak (2017). All rights reserved.

In such techniques, the choice of the GPE is crucial: any sequential design method will only work well if the chosen GPE is a good description of the true model function. This issue is exemplarily highlighted in an earlier study by Sinsbeck and Nowak (2017). In that study, a Bayesian inverse problem was solved using an SDoE method. To test the sensitivity, GPEs with different covariance functions were compared in their performance. Figure 1 shows the errors in the target quantity (i.e., the posterior probability of model parameters) over the number of model evaluations. Each data line corresponds to a different GPE. We can observe that, with the right GPE, a small error can be achieved within 30 model evaluations (GPE 1 and GPE 2). A poorly chosen GPE, at the same time, leads to an error that does not even decrease within the first 30 model evaluations (GPE 6). In summary, the performance of sequential design methods is highly sensitive to the choice of the GPE.

A GPE is fully described by its mean and covariance functions, so by *choice of GPE* we actually mean the choice of a parametric function for both mean and covariance and the choice of the corresponding parameter values. Examples for parametric functions are a mean of constant zero and a covariance of Matérn type (e.g., Handcock and Stein, 1993; Stein, 1999; Diggle et al., 2003; Minasny and McBratney, 2005; Diggle and Lophaven, 2006). In the following we will refer to the parameters of the GPE as *hyperparameters*. The Matérn covariance function has three hyperparameters: a standard deviation, a correlation length and a smoothness parameter.

The most straight-forward approach for obtaining the hyperparameters is splitting the whole computing procedure into two phases: In the first phase, the so-called exploratory phase, the model function is evaluated on a space-filling set of design points, the so-called initial design. From these model responses, the hyperparameters are estimated, for example via the maximum likelihood method. The initial set of design points is specifically chosen for exploring the parameter space and for estimating the hyperparameters. Then, in the second phase, the actual sequential design is carried out: using the found hyperparameters, the corresponding GPE is used to solve the actual problem. Design points in this second phase are chosen by the sequential sampling strategy and are specifically selected to solve the problem at hand. Since SDoE methods are mostly used in problems that require local accuracy, these new design points are typically not space-filling, but concentrate in problem-driven areas of interest.

Usually, hyperparameters are iteratively re-estimated in the second phase in order to update the GPE using the evaluated design points (see e.g., Bect et al., 2012). Yet, the two phases are typically separate. Our hypothesis is that such a two-phase procedure is disadvantageous in two ways: first, it makes the procedure unnecessarily complicated, and second, it requires more computer time than necessary:

1. The exploratory phase complicates the use of the method by introducing more choices: how many model evaluations should be spent in the first sampling phase (Ranjan et al., 2008; Loeppky et al., 2009; Bect et al., 2012), and where should the model be sampled?
2. The exploratory phase requires computer time. It seems wasteful to first select inputs with the sole purpose of finding the hyperparameters and non-adaptive exploration and then to select some more with the sole purpose of solving the problem at hand.

Computational resources are always limited, and various approaches were proposed on how to optimally allocate them to achieve both global exploration and local exploitation (e.g., Sóbester et al., 2005; Chen et al., 2016). The purpose of this article is to investigate whether there are synergetic effects between the iterative structure of SDoE methods and the task of estimating hyperparameters that can be used to alleviate the issue of budget allocation. Can we find appropriate hyperparameters dynamically without an exploratory phase, i.e., while the GPE is already in use for solving the problem at hand? If we can do so, then we might be able to eliminate the exploratory phase and thereby make sequential design methods both faster and easier to use.

Note that the exploratory phase can only be eliminated under one additional condition: often, an exploratory phase has a second purpose besides estimating hyperparameters, and that is guaranteeing that the parameter domain is explored to a certain extent. This can be useful if the problem is multimodal. For example, when a Bayesian optimization method is applied to an optimization problem with local optima, then the exploratory phase can help find the global optimum. If we intend to eliminate the exploratory phase, then we have to make sure that the sequential sampling strategy itself strikes a proper balance between exploration and refinement. Whether this is the case depends very much on the chosen method, and is out of scope of this article. Here, we will only investigate whether hyperparameters can be estimated without exploratory phase.

As many models in hydro(geo)logy react monotonously to changes in parameters, the resulting inverse problems tend to be unimodal. Therefore, our research is highly relevant, in specific, for hydro(geo)logical models. For such types of problems, tailored approaches exist that, e.g., include monotonicity information into the GPE (Riihimäki and Vehtari, 2010; López-Lopera et al., 2018). Yet, an explicit consideration of whether an expensive exploration phase is always necessary was missing. We fill this gap, proposing our method specifically to users in the applied sciences (see e.g., Erickson et al., 2018; Gramacy, 2020) like hydro(geo)logy. By estimating hyperparameters dynamically under the condition above, our method releases modelers from potential drawbacks associated to hyperparameter estimation and therefore fosters easy access to using GPE in applied modeling.

As mentioned earlier, SDoE methods exist for a range of problem types, such as optimization, contour estimation, estimation of failure probability, and so on. To keep the scope of our article manageable, we restrict the numerical experiments to the problem type of solving Bayesian inverse problems. Whether the results can be generalized to the other problem types, is discussed at the end of the article.

The article is structured as follows. In section 2, we summarize the classical approach that includes an exploratory phase. In section 3, we discuss the changes required in the algorithm when hyperparameters are estimated without the exploratory phase. Section 4 shows numerical examples from the area of hydro(geo)logy to demonstrate the exploratory-phase-free estimation of hyperparameters. In section 5, we investigate, whether the previous results allow us to eliminate the exploratory phase altogether. Finally, section 6 provides a discussion and a summary. In the Supplementary Material, we provide additional numerical examples.

## 2. Sequential Design of Computer Experiments With Exploratory Phase

In this section, we describe the classical case, in which a sequential design method is used in combination with an exploratory phase. First, we introduce Gaussian process emulators (GPE) as the main tool. Second, we outline the general structure of sequential design of experiments (SDoE) methods. Third, we explain the purpose of the exploratory phase. And fourth, we recap the sequential design method for solving Bayesian inverse problems. The first three sections are general and independent of the problem type at hand. Only the last section is specific to solving Bayesian inverse problems.

### 2.1. Gaussian Process Emulators

This section provides a very short introduction to our notation for Gaussian process emulators. More details can be found in the literature (e.g., Sacks et al., 1989; Kitanidis, 1997; Stein, 1999; Kennedy and O'Hagan, 2001; Higdon et al., 2004; O'Hagan, 2006; Rasmussen and Williams, 2006).

#### 2.1.1. Conditioning

Let $\Omega \subseteq {\mathbb{R}}^{n_{p}}$ be an input domain (e.g., a model's parameter space) and let $u:\Omega \to {\mathbb{R}}^{n_{o}}$ be a model function mapping input parameters to some model output. The function *u* is usually given in the form of some simulation software, and we assume that each evaluation is computationally expensive. To emulate *u*, we use a Gaussian process emulator *U*_0_ ~ GP(*m*_0_, *k*_0_) with mean function *m*_0_ and covariance function *k*_0_.

Now, let *x*_1_, …, *x*_*n*_ ∈ Ω be a number of points in the input domain, at which the output *u*(*x*_1_), …, *u*(*x*_*n*_) is observed. Conditioning the (prior) GPE *U*_0_ to these *n* observations leads to a conditioned emulator *U*_*n*_, which itself is a GPE: *U*_*n*_ ~ GP(*m*_*n*_, *k*_*n*_). To compute the conditional mean and covariance function, we define the residual vector *r* = [*u*(*x*_1_) − *m*_0_(*x*_1_), …, *u*(*x*_*n*_) − *m*_0_(*x*_*n*_)] and the covariance matrix *Q* by [*Q*]_*ij*_ = *k*(*x*_*i*_, *x*_*j*_), and for a point *x* ∈ Ω, we define the covariance vector *q*(*x*): = [*k*(*x*_1_, *x*), …, *k*(*x*_*n*_, *x*)]. We then obtain

(1) $$\;\begin{array}{ll} \;m_{n}\left(x\right) & =m_{0}\left(x\right)+q\left(x\right)Q^{-1}r^{⊤},\\ k_{n}\left(x,x^{\prime }\right) & =k_{0}\left(x,x^{\prime }\right)-q\left(x\right)Q^{-1}q{\left(x^{\prime }\right)}^{⊤}, \end{array}$$

which is well-known in hydro(geo)logy under the name of Kriging, yet applied to a model output as a function of its parameters, not to some parameters as a function of space.

If the model output is multivariate (*n*_*o*_ > 1), then we construct an independent GPE for each output component.

#### 2.1.2. Mean and Covariance Functions

The results of this work hold for GPEs with any kind of mean and covariance function. This section summarizes the mean and covariance functions used in the numerical examples of this article.

The mean function used for all GPEs in this article is the constant zero mean: *m*_0_(*x*) = 0. Alternatively, the mean could also be set as unknown which is sometimes referred to as ordinary Kriging, as polynomial function (universal Kriging) or any other function like a simplified mechanistic model (e.g., Machac et al., 2018).

The two types of covariance function used in this article are the squared exponential covariance function (e.g., Santner et al., 2003; Rasmussen and Williams, 2006) and the Matérn covariance function (e.g., Handcock and Stein, 1993; Stein, 1999; Diggle et al., 2003; Minasny and McBratney, 2005; Diggle and Lophaven, 2006; Rasmussen and Williams, 2006) with the former being a special case of the latter. These are based on a normalized distance *d*(*x, x*′) between two points *x* and *x*′, which can be expressed as $d\left(x,x^{\prime }\right)=∥L^{-1}\left(x-x^{\prime }\right){∥}_{2}$. Here, the matrix *L* contains correlation length information about the input domain. If the model function is assumed to be isotropic, then *L* = λ*I*, i.e., matrix *L* is the identity matrix *I* multiplied with a scalar correlation length parameter λ. If the model function is assumed to be anisotropic with axis-aligned anisotropy, then *L* is a diagonal matrix with the individual correlation length parameters on the diagonal: *L* = diag(λ_1_, λ_2_, …, λ_*n*_*p*__). Non-axis-aligned anisotropic cases are possible, too, but will not be considered in this article.

With a distance *d*, the squared exponential (SE) covariance and Matérn covariance have the following form:

$$\begin{array}{l} k_{\text{SE}}\left(x,x^{\prime }\right)=\sigma^{2}\exp\left[-\frac{d{\left(x,x^{\prime }\right)}^{2}}{2}\right]\\ k_{\text{Matérn}}\left(x,x^{\prime }\right)=\sigma^{2}\frac{2^{1-\nu }}{\Gamma \left(\nu \right)}\cdot {\left(\sqrt{2\nu }d\left(x,x^{\prime }\right)\right)}^{\nu }K_{\nu }\left(\sqrt{2\nu }d\left(x,x^{\prime }\right)\right). \end{array}$$

Here, σ^2^ is a variance parameter, and ν is a smoothness parameter (only present in the Matérn covariance function). Furthermore, Γ denotes the gamma function and *K*_ν_ denotes the modified Bessel function of the second kind. Thereby, $k_{\text{SE}}\left(x,x^{\prime }\right)$ is the limit of $k_{\text{Matérn}}\left(x,x^{\prime }\right)$ for ν → ∞.

We call the parameters that define the mean and covariance function *hyperparameters* (as opposed to the elements of Ω, which are called input parameters) and denote them as θ. To indicate the dependence on the hyperparameters, the mean and covariance functions will be called *m*_θ_ and *k*_θ_.

### 2.2. Sequential Design of Computer Experiments: General Structure

Comparing SDoE methods from the literature (Kushner, 1964; Jones et al., 1998; Williams et al., 2000; Bichon et al., 2008; Ranjan et al., 2008; Picheny et al., 2010; Bect et al., 2012; Mockus, 2012; Balesdent et al., 2013; Shahriari et al., 2016; Sinsbeck and Nowak, 2017; Ginsbourger, 2018), we find that they all share the same general structure. They all revolve around a quantity of interest QoI = *q*(*u*) that depends on a computationally expensive function *u*. This quantity of interest can, for example, be the location of the minimum of *u* or a failure probability or a posterior distribution. To obtain an accurate estimate of *q*(*u*) while keeping the number of function evaluations of *u* small, the function *u* is emulated by a GPE *U*_0_. During the SDoE, the algorithm selects a sequence of *design points* *x*_1_, *x*_2_, …, *x*_*n*_ ∈ Ω. These are the parameter vectors for which the model function *u* is evaluated. In the beginning, the first design point *x*_1_ is selected and the function *u* is evaluated at that point, so we obtain the first observation *u*(*x*_1_). Then, the emulator *U*_0_ is conditioned to the observation *u*(*x*_1_). Based on the conditioned emulator *U*_1_, the next design point *x*_2_ is selected, the function *u* is evaluated and the emulator *U*_0_ is conditioned to *u*(*x*_1_) and *u*(*x*_2_). This procedure is repeated, with more and more design points being added, until an exit condition is met. After the final iteration, the emulator *U*_0_ is conditioned to all observations *u*(*x*_1_), *u*(*x*_2_), …, *u*(*x*_*n*_), and an estimate of *q* is computed based on the final conditioned emulator *U*_*n*_. The computational rule to make this estimate for *q* based on an emulator *U* is called an *estimator* (Bect et al., 2012) and is denoted by $\hat{q}\left(U\right)$.

The sampling strategy for selecting the next design point is typically based on an *acquisition function* α. The acquisition function is a function of *x* and of the conditioned emulator from the previous iteration *U*_*i* − 1_. Its minimum defines the next design point:

$$x_{i}=\underset{x\in \Omega }{\text{arg min}}\alpha \left(x,U_{i-1}\right).$$

The specific formula for the acquisition function depends on the problem at hand.

**Figure 2:**
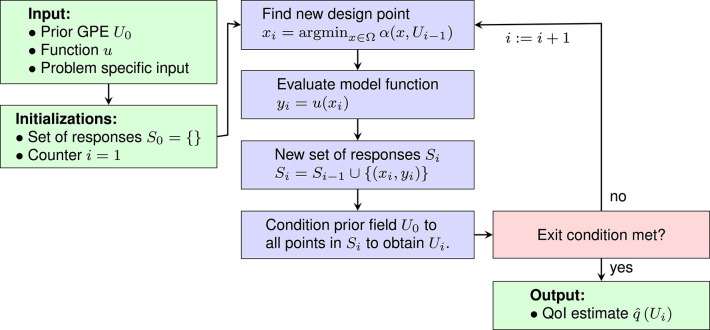
General structure of sequential design of experiments methods.

Figure 2 is a flow chart showing the general structure of SDoE methods. Note that the GPE *U*_0_ is an input to the method, so *U*_0_ with its hyperparameters needs to be selected before the sequential design can be started. This is the purpose of the exploratory phase.

### 2.3. Exploratory Phase

Hyperparameters are usually estimated initially in a separate sampling phase, called the *exploratory phase* (e.g., Jones et al., 1998; Williams et al., 2000; Bichon et al., 2008; Ranjan et al., 2008; Picheny et al., 2010; Bect et al., 2012; Balesdent et al., 2013). In this phase, the function *u* is sampled on a space-filling set of design points, the so-called *initial design*. The initial design is typically chosen randomly (e.g., Bichon et al., 2008; Wang and Jegelka, 2017), quasi-randomly [e.g., latin hypercube sampling (Jones et al., 1998; Williams et al., 2000; Ranjan et al., 2008; Picheny et al., 2010; Bect et al., 2012; Balesdent et al., 2013)] or by hand (e.g., corners of the domain Picheny et al., 2010). Using the model responses on the initial design, the hyperparameters are found via maximum-likelihood (ML) estimation or, if a prior exists, via maximum-a-posteriori (MAP) estimation. Some authors suggest to validate the hyperparameters to assure generalizability (Jones et al., 1998; Forrester et al., 2008; Kleijnen, 2018). The corresponding GPE is then conditioned to the model responses on the initial design to form the prior GPE *U*_0_, see Figure 2.

While often not stated explicitly, the exploratory phase has a second purpose besides estimating hyperparameters: guaranteeing a minimum degree of exploration. This can be useful, if the acquisition function on its own overemphasizes refinement over exploration. An exploratory phase in the beginning can compensate for this to some extent. This means that, even if we can find appropriate hyperparameters without an exploratory phase, we might not be able to eliminate it due to its other purpose.

The iterative re-estimation of hyperparameters is then conducted during the subsequent sequential design phase.

### 2.4. Sequential Design for Bayesian Inverse Problems

In this section we briefly introduce our notation for Bayesian inverse problems (Tarantola, 2005; Stuart, 2010) and present the acquisition function and estimator used to solve such problems sequentially.

We consider a model function $u:\Omega \to {\mathbb{R}}^{n_{0}}$ as before. Additionally we define a random variable *X* with values in Ω that describes the uncertainty in the model input. We assume that *X* has a multivariate probability density function (pdf) π. Next, we assume an additive error model. A measurable quantity is defined by *Z* = *u*(*X*)+ ε, where ε is a random error with (multivariate) pdf *p*_ε_. Once a measurement of *Z* is obtained (*Z* = *z*), we are interested in computing the posterior pdf π′ of *X* conditional to the observation *Z* = *z*. According to Bayes' theorem we find:

$$\pi^{\prime }\left(x\right)=\frac{L\left(x\right)\pi \left(x\right)}{\int_{\Omega }L\left(x_{0}\right)\pi \left(x_{0}\right)\text{d}x_{0}}.$$

Here, *L* is the likelihood of measuring *Z* = *z* if *X* = *x*. It can be computed as *L*(*x*) = *p*_ε_(*z* − *u*(*x*)). The main difficulty in computing the posterior pdf π′ lies in the likelihood function. Each evaluation of the likelihood function requires one model evaluation.

An SDoE method for solving such Bayesian inverse problems was developed by Sinsbeck and Nowak (2017). The method has the general structure as described in section 2.2, so for brevity we only provide the formula for the estimator $\hat{q}\left(U\right)$ and the acquisition function α. Details and derivations can be found in the original article.

The quantity of interest in this case is the likelihood: *q* = *L*. To write the formula for the estimator $\hat{q}\left(U\right)$, we first expand the previous notation of the likelihood, and add the model as a second argument: *L*(*x*|*u*) = *p*_ε_(*z* − *u*(*x*)). Replacing the model *u* by an emulator *U* leads to a random likelihood *L*(*x*|*U*). The estimator that is optimal in the average *L*_2_-sense is the average likelihood

(2) $$\begin{array}{r} \hat{q}\left(U\right)={\text{E}}_{U}\left[L\left(\cdot |U\right)\right]. \end{array}$$

For the formulation of the acquisition function, we first define a loss which measures the uncertainty about the likelihood estimate:

(3) $$\begin{array}{r} l\left(U\right)=\int_{\Omega }\text{Var}\left[L\left(x|U\right)\right]\pi \left(x\right)\text{d}x. \end{array}$$

Next, let *U*_(*x, y*)_ be the GP we obtain after training *U* to the additional point (*x, y*) with *y* = *u*(*x*). Ideally, we would like to select a design point *x*, such that the loss *l*(*U*_(*x, y*)_) is minimized. The model response *y*, however, is yet unknown. Fortunately, it has a known distribution according to the current GP: *Y* = *U*(*x*). Therefore, we define the acquisition function as the expected loss:

$$\alpha \left(x,U\right)={\text{E}}_{Y}\left[l\left(U_{\left(x,Y\right)}\right)\right].$$

In words, this acquisition function represents the residual variance in the likelihood estimate after the next model evaluation, averaged over the possible yet unknown model responses.

The definition of this acquisition function α does not require the random field *U* to be Gaussian. It is generally defined for both Gaussian and non-Gaussian random fields. The numerical computation of α, however, is only tractable in the Gaussian case, because then conditioning is linear, see Equation (1), and the conditional variance is independent of the model response. In the general non-Gaussian case, the computation of α is infeasible with current means.

## 3. Hyperparameter Estimation Without Exploratory Phase

Hyperparameters are unknown at the beginning of the algorithm. Therefore, we will model them as random variables. With this change, our GPE *U*_0_ becomes a non-Gaussian random field, because a mixture of Gaussian distributions with different means and variances is not Gaussian anymore. Therefore, we need to revisit all steps in the algorithm that involve the random field. These are (i) conditioning the random field to model evaluations, (ii) computing the acquisition function α and (iii) computing the estimator $\hat{q}$. These steps will be addressed in the following sections.

### 3.1. Hyperparameters Are Random

We now consider the case where the modeler has decided to use a mean function and covariance function of a certain type, but does not know how to select the hyperparameters. Instead of selecting fixed numbers, we make the hyperparameters random themselves by assigning them a prior distribution (e.g., Higdon et al., 2008; Snoek et al., 2012; Hernández-Lobato et al., 2014). We summarize all of the hyperparameters in a random vector Θ with pdf *p*_Θ_. Specific realizations of the hyperparameters will use the lower-case θ. To denote the dependence of the mean and covariance functions on the hyperparameters, we write *m*_θ_ and *k*_θ_. Furthermore, we denote the Gaussian subfield of *U* with any given set of hyperparameters θ as *U*^(θ)^.

If hyperparameters are completely unknown, it is also possible to infer them with prior-free methods, such as maximum-likelihood estimation. An argument for doing so is that finding a prior itself is an additional modeling step that potentially can go wrong. After all, it is not immediately clear why selecting a prior is easier in practice than selecting specific hyperparameter values. With the following subsections, we argue, why it is beneficial to select a hyperparameter prior, and in the numerical experiments, we demonstrate that it is possible to select an appropriate prior according to some simple rules of thumb.

#### 3.1.1. The Role of the Hyperparameter Prior

Here, the prior is not meant to be a Bayesian expression of belief (Jaynes, 2003). We rather understand the prior as a mathematical tool to avoid extreme values that might lead to numerical problems. Such a prior is called a *regularizing* prior (Gelman et al., 2017). At early stages in the iterative procedure, when the model has only been evaluated a few times, the prior is meant to center the hyperparameters on some plausible values. With more model evaluations available, the hyperparameter likelihood will become more informative and the prior is expected to play a diminishing role. To achieve this behavior, we select a prior with a relatively large variance.

#### 3.1.2. Finding a Hyperparameter Prior

In this section, we provide a possible practical rule for finding an actual hyperparameter prior. In the numerical experiments in section 4, we will show that this rule is good enough to provide good results. Of course, other options are possible, too, such as Gamma priors (e.g., Higdon et al., 2008; Hernández-Lobato et al., 2014).

All of the hyperparameters considered are strictly positive, so we describe them with a log-normal prior. To find the distribution parameters μ and σ^2^ of the log-normal distribution for each hyperparameter, we think in terms of (soft) upper and lower bounds (*b*_lower_, *b*_upper_) and set these to be the ±2σ-values in log-space:

$$\begin{array}{l} \mu +2\sigma =\log\left(b_{\text{upper}}\right)\\ \mu -2\sigma =\log\left(b_{\text{lower}}\right). \end{array}$$

With this construction, the two soft bounds roughly correspond to the 5 and 95%-percentiles of the log-normal distribution. For the individual hyperparameters, we suggest the following upper and lower bounds:

- Correlation length *l*. The length of the input domain can be expected to be a safe upper bound for the correlation length. A GPE with that correlation length is essentially a constant function, so larger values do not seem to be meaningful. As a lower bound, we suggest using a value around 1/100th to 1/1.000th of the domain length. Expecting a correlation length smaller than that would mean that the function *u* has some very high frequency effects. That, in turn, would mean that meaningful interpolation with few function evaluations is not possible anyways. In those cases, GPE-based methods are not expected to work efficiently and it is probably better to use a different approach entirely.
- Variance σ^2^. To find bounds for the variance, we can use the measurement value *z* as an orientation, because it should be a plausible realization of the GPE. Since we only consider random fields with a zero-mean for simplicity, we use the raw second moment of *z* as an anchor point. We multiply this anchor point by at least a factor of 10 and $\frac{1}{10}$ to obtain an upper and lower bound, respectively.
- Smoothness parameter ν. We set the lower bound to 0.5. This is the well-known edge case, where the Matérn covariance becomes an exponential function (e.g., Minasny and McBratney, 2005). At this value or below, realizations of the GPE are not differentiable, while most models are differentiable in most parts of the parameter domain. For the upper bound, we suggest the value 10. While it is desirable to allow large ν values to describe very smooth functions, very large values lead to numerical problems when evaluating the covariance function. Here, the upper bound of 10 can be understood as a regularization. The numerical examples will show that this upper bound is good enough.
  In cases where the model function is known to be infinitely smooth, it is suggested to use a squared-exponential covariance function and avoid the hyperparameter ν altogether. Thereby, potential difficulties in hyperparameter estimation can be reduced (see e.g., Kaufman and Shaby, 2013).

### 3.2. Conditioning Non-gaussian Random Fields

A GPE with random hyperparameters becomes a *conditionally Gaussian random field* (Kitanidis, 1997). It is still a random field, but generally a non-Gaussian one. Conditional to a specific hyperparameter vector, it becomes Gaussian again. Such a non-Gaussian random field can also be thought of as an uncountable Gaussian mixture (Hennig and Schuler, 2012).

In the sequential design method, finding the hyperparameters is a secondary goal that only serves the primary goal of solving the problem at hand. Therefore, we do not need to find the hyperparameters in a deterministic sense. We may retain uncertainty about the hyperparameters as long as the effect on the estimated quantity of interest $\hat{q}$ is small. This leads us to two possible ways of handling uncertain hyperparameters (Shahriari et al., 2016):

The first way is to consider them as what they are—random variables (e.g., Osborne et al., 2009; Brochu et al., 2010; Snoek et al., 2012; Garnett et al., 2014). When the model function is evaluated at a number of points, then these observations (*x*_*i*_, *u*(*x*_*i*_)) define a marginal posterior distribution for the hyperparameters. Usually there is no analytical expression for this distribution, but its density function can be evaluated pointwise, so we can approximate the distribution with a sample from a Markov Chain Monte Carlo (MCMC) method (Hastings, 1970). A posterior sample of the hyperparameters is useful in two ways :

1. It allows us to approximate the conditioned random field as a countable Gaussian mixture model. To do so, we draw a posterior sample of the hyperparameters, construct a GPE with each realization and then condition each GPE to the model evaluations.
2. With such a sample, we can approximate any expected value over the hyperparameters (𝔼_Θ_[*f*(Θ)], where *f* can be any arbitrary function). In the following, we regard such integrals over Θ as feasible (as long as the inner function *f* is easy to compute).

The second way of handling uncertain hyperparameters is to make a point estimate of them, typically the MAP estimate θ_MAP_ or ML estimate θ_ML_. While this approach neglects the uncertainty about the hyperparameters, it has two advantages. First, it reduces the random field to a Gaussian one and, second, it does not require any sampling because the point estimates can be found by optimization.

### 3.3. Computing the Acquisition Function

In this section, we present and discuss different methods for computing the acquisition function, when hyperparameters are random. The first method is to apply the acquisition function to the non-Gaussian random field. Since this is not always possible, we introduce three different simplified approaches: *dynamic MAP estimation, criterion averaging*, and *Gaussian process linearization*. These simplifications only require us to evaluate the acquisition function on Gaussian random fields. The first two simplified approaches are established and can be found in the literature (see references in the following sections) while the third one, Gaussian process linearization, is novel and presented here for the first time.

#### 3.3.1. Computing or Approximating the Full Acquisition Function

Conceptually the most straightforward way of handling unknown hyperparameters is to incorporate them into the random field and simply compute the acquisition function α(*x, U*) on the resulting non-Gaussian random field *U*. Whether this is tractable, depends on the acquisition function.

In Bayesian Optimization, many acquisition functions have a very simple form. Examples are the probability of improvement, the expected improvement and Thomson-sampling (Thompson, 1933; Kushner, 1964; Shahriari et al., 2016). Applying these to a conditionally Gaussian random field is possible either analytically or with a Monte-Carlo (MC) approximation (more on that below in section 3.3.3).

However, there are also acquisition functions where this is not possible, most notably so called information-based acquisition functions (e.g., Villemonteix et al., 2009; Bect et al., 2012; Hennig and Schuler, 2012; Balesdent et al., 2013; Hernández-Lobato et al., 2014; Sinsbeck and Nowak, 2017; Wang and Jegelka, 2017). These acquisition functions quantify how much information is gained by conditioning the emulator to a certain model response. But since the future model response is not observed yet, they consider the average over possible model responses. This structure makes it infeasible to compute or approximate such acquisition functions on a random field that is only *conditionally* Gaussian.

#### 3.3.2. Dynamic MAP Estimation

In this approach, we dynamically estimate hyperparameters via their maximum-a-posteriori (MAP) estimate. This approach is a Bayesian variant of the common re-estimation of hyperparameters, specified by two characteristics: first, a new hyperparameter estimate is made in each iteration. Second, there is no exploratory phase, only those evaluations of *u* are used that are made within the sequential design method anyway. This means that, in the first iteration, the hyperparameters are determined using only the hyperparameter prior. Then, in the second iteration, the prior is combined with the likelihood from the first evaluation. In the third iteration, the first two evaluations are used, and so on. If we made a maximum-likelihood estimate instead of a MAP estimate, then we would obtain a prior-free method.

In this context, note that the re-estimation of hyperparameters might require additional considerations. For example, Picheny et al. (2010) argue that re-estimating hyperparameters can be problematic: design points are specifically chosen, such that the model response is close to the measurement (or, in the case of their article, close to a threshold). That way, the model function's variance might be underestimated or the hyperparameter estimate might be biased in other, unforeseen ways. The article, however, does not provide an example of this problem and in the current study we did not encounter any such problems.

#### 3.3.3. Acquisition Function Averaging

A second approach is to average the acquisition function over the hyperparameters to obtain the so-called *integrated* or *marginalized* acquisition function (e.g., Snoek et al., 2012; Hernández-Lobato et al., 2014):

$$\alpha_{\text{average}}\left(x,U\right):={\text{E}}_{\Theta }\left[\alpha \left(x,U^{\left(\Theta \right)}\right)\right],$$

where the hyperparameters Θ are distributed according to the most current hyperparameter posterior. If the original acquisition function α itself is a probability or an expected value over the random field *U*, then, by the law of total probability/total expectation, it follows that α_average_ = α. Two examples for such acquisition functions are the probability of improvement and the expected improvement in Bayesian optimization (Snoek et al., 2012). In most cases, however, α_average_ will be different from α. The acquisition function for Bayesian inverse problems, as presented in section 2.4, is an example for this, because it internally conditions the GPE to possible model responses, which implies different hyperparameter posteriors. Therefore, the hyperparameters cannot be averaged out analytically as α_average_ = α.

As argued in section 3.2, the computation of an expected value over Θ is feasible, if the computation of the inner term is feasible. Here, this is the case, because for any realization θ, the emulator *U*^(θ)^ is Gaussian, and we assume the acquisition function to be computable on GPEs. Besides the obvious MC-estimate of the expected value (Brochu et al., 2010; Snoek et al., 2012), there also exist approximation-based approaches (e.g., Garnett et al., 2014).

#### 3.3.4. Gaussian Process Linearization

A third simplification is to replace the non-Gaussian random field by a GPE with the same mean and covariance function. Given the most current distribution of the hyperparameters Θ, the overall mean and covariance function of *U* can be computed as

$$\begin{array}{l} \;m\left(x\right)=\text{E}\left[U\left(x\right)\right]={\text{E}}_{\Theta }\left[m_{\Theta }\left(x\right)\right]\\ k\left(x,x^{\prime }\right)=\text{Cov}\left[U\left(x\right),U\left(x^{\prime }\right)\right]\\ \;={\text{E}}_{\Theta }\left[k_{\Theta }\left(x,x^{\prime }\right)\right]+{\text{E}}_{\Theta }[(m_{\Theta }\left(x\right)\\ \;-m\left(x\right))(m_{\Theta }(x^{\prime })-m(x^{\prime }))]. \end{array}$$

These can numerically be approximated via an MC-estimate after drawing an MCMC sample of the hyperparameters θ, see section 3.2. Recall that *m*_θ_ and *k*_θ_ denote the mean and covariance function of *U*^(θ)^. Next, we define the linearized GPE as *U*_linearized_ ~ GP(*m, k*) and obtain the acquisition function

$$\alpha_{\text{linearized}}\left(x,U\right):=\alpha \left(x,U_{\text{linearized}}\right).$$

We call this method linearization, because it allows us to use the linear conditioning equations, see Equation (1). The second term on the right-hand side of the covariance function translates hyperparameter uncertainty into a larger covariance. That way, the hyperparameter uncertainty enters the acquisition function in a linearized way.

Furthermore, Gaussian process linearization is the only approach that is, in principle, applicable to random fields that are not even conditionally Gaussian, such as the square of a GPE (compare Zinn and Harvey, 2003).

### 3.4. Estimating the Quantity of Interest

Similarly to the acquisition function, handling the estimator $\hat{q}$ depends on the type of problem at hand. In Bayesian optimization, the estimator simply returns the design point with the lowest value in the objective function. This does not require any change if the emulator is non-Gaussian. In most problem types, such as in estimation of failure probabilities and in Bayesian inverse problems, the quantity of interest is a probability or an expected value, so we can apply the law of total probability/total expectation and obtain

$$\hat{q}\left(U\right)={\text{E}}_{\Theta }\left[\hat{q}\left(U^{\left(\Theta \right)}\right)\right].$$

This expression can, again, be computed with an MC-estimate.

Alternatively, if the hyperparameters are handled as a point estimate anyways, e.g., θ_MAP_, then we could approximate

$$\hat{q}\left(U\right)\approx \hat{q}\left(U^{\left(\theta_{\text{MAP}}\right)}\right).$$

If the hyperparameter posterior is very much concentrated, then this can be a good approximation.

## 4. Numerical Experiments

With the numerical experiments in this section, we assess whether hyperparameter can be estimated without an exploratory phase (as mentioned earlier, this does not yet mean that we can eliminate the exploratory phase, which is tested in section 5). To evaluate the performance of our methods, we compare them with *random guessing* and the hypothetical case *miracle*, where hyperparameters are already known.

Random guessing assumes that a hyperparameter prior is available as described in section 3.1.2. From this prior, we randomly draw hyperparameters and run the SDoE method. This approach is supposed to show that just knowing the prior is not enough. If random guessing performs poorly and the exploratory-phase-free methods perform much better, then this confirms that the hyperparameter prior did not contain hidden information for solving the problem and, furthermore, that finding a prior by hand is much easier in practice than finding the hyperparameters themselves.

In the case miracle, we use hyperparameters that are expected to be close to optimal. These were found by maximum likelihood estimation from various samples (both space filling and from the posterior of the inverse problem). Then, the hyperparameters that performed best were selected by hand. Since these hyperparameters are based on extensive sampling of the model functions, they use information that is not available in practice. Therefore, they serve as a “best-case” benchmark. If the exploratory-phase-free methods perform similarly to the miracle case, then we conclude that they successfully found good enough hyperparameters.

As mentioned before, we will only test these exploratory-phase-free approaches in an SDoE method for Bayesian inverse problems. Whether the results are transferable to other problem types will be discussed below in section 6.1.

To compare the various approaches, we perform two experiments. In the first experiment, we provide a general overview of the performance of the different approaches. In the second experiment, we examine a slightly higher-dimensional problem with a larger number of hyperparameters. To provide more evidence for the results of this work, two more experiments are reported as Supplementary Material to this article.

All experiments are written in python. MAP estimates were found using the BFGS method (a local optimizer in the standard scipy-library). For MCMC sampling , the package *Emcee* (Foreman-Mackey et al., 2013) was used. It implements the “Affine Invariant Markov chain Monte Carlo Ensemble sampler” (Goodman and Weare, 2010).

The research code for all of the results is available online^1^.

### 4.1. Experiment 1: Source Identification

In the first experiment, we consider a problem that has been used as a test case in a number of publications on inverse problems (e.g., Marzouk et al., 2007; Li and Marzouk, 2014; Sinsbeck and Nowak, 2017). It consists of the two-dimensional diffusion equation on the unit square [0, 1]^2^:

$$\frac{\partial c\left(\xi ,t\right)}{\partial t}=\nabla^{2}c\left(\xi ,t\right)+s\left(\xi \right).$$

Here, *t* denotes time and ξ denotes coordinates in the spatial domain (the unit square). Furthermore, *c*(ξ, *t*) is concentration and *s*(ξ) is a source term, given as

$$s\left(\xi \right)=\frac{s_{0}}{2\pi h^{2}}\exp\left(\frac{-|X-\xi {|}^{2}}{2h^{2}}\right),$$

where *X* is the location of the source. Identifying *X* from concentration measurements is the purpose of this inverse problem. We assume a uniform prior for the source location $X\sim U\left({\left[0,1\right]}^{2}\right)$. The other variables are deterministic with *s*_0_ = 2 and *h* = 0.05, and the initial condition is set to *c*(ξ, 0) = 0. On the boundary, we impose no-flux boundary conditions.

At two time steps *t*_1_ = 0.1 and *t*_2_ = 0.2, the concentration *c* is measured on a 3 × 3 grid spanned by the corners of the domain, resulting in a total of 18 measurements, so the model function *u*:ℝ^2^ → ℝ^18^ maps a two-dimensional source location to 18 concentration values. For each measurement, *i* = 1, …, 18, an additive and independent normally distributed error $\varepsilon_{i}\sim N\left(0,0.1^{2}\right)$ is assumed. A virtual measurement is generated by setting the source position *X* = (0.25, 0.25), running the forward simulation on a fine grid of resolution 2048 × 2048, and adding random noise. For the solution of the Bayesian inverse problem itself, the diffusion equation is solved on a 512 × 512 grid. The two grid resolutions are different to avoid a so-called inverse crime (Kaipio and Somersalo, 2007) (this is when the same model is used for data generation and inverse, resulting in a particularly well-posed inverse problem).

As a stochastic grid for the input parameter *X*, we choose a regular 51 × 51 grid. This grid is used within the sequential design for computing the stochastic integrals and also for optimizing the acquisition function (by complete enumeration on this grid). Furthermore, this grid is used for computing the reference posterior and errors between approximate posteriors and the reference posterior.

The model function is emulated by a GPE of Matérn type with zero mean. The 18 output components of the model are assumed to be independent and identically distributed, meaning that we use the same hyperparameters for all 18 components. For defining the hyperparameter prior, we use the procedure presented in section 3.1.2. The soft bounds are summarized in Table 1, in the first row under the name “default.”

**Table 1:** Soft bounds for the hyperparameter prior.

	**Corr. length** **λ**		**Variance** **σ**^**2**^		**Smoothness** **ν**	
	***b*_lower_**	***b*_upper_**	***b*_lower_**	***b*_upper_**	***b*_lower_**	***b*_upper_**
Default	10^−2^	10^0^	10^−2^	10^1^	0.5	10
Wide upper & lower	10^−3^	10^1^	10^−3^	10^2^	0.5	10
Wide upper	10^−2^	10^1^	10^−2^	10^2^	0.5	10
Narrow	10^−2^	10^−1^	10^−2^	10^−1^	0.5	10

*These bounds roughly correspond to the 5 and 95%-percentiles of the hyperparameters, see section 3.1.2*.

#### 4.1.1. Exploratory-Phase-Free Methods vs. Random Guessing and Miracle

**Figure 3:**
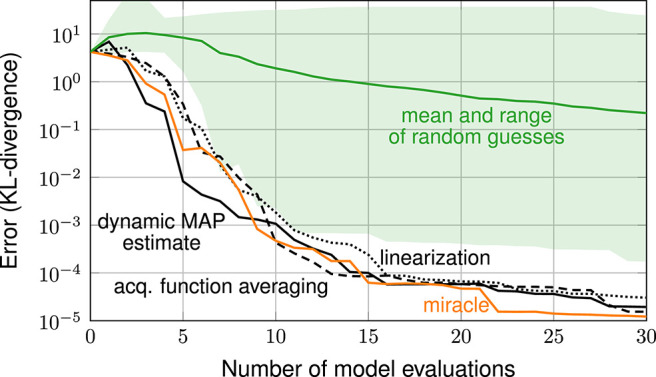
Error plot: exploratory-phase-free methods vs. random guessing and case miracle.

Figure 3 shows the error in estimating the posterior over the number of iterations. The error is measured in terms of the KL-divergence between reference posterior and estimated posterior (discretized on the 51 × 51 stochastic grid). The plot shows the error resulting from the various exploratory-phase-free methods, from random guessing and from the miracle case (with hyperparameters λ ≈ 0.34, σ^2^ ≈ 0.034 and ν ≈ 5.5 found from a large space-filling sample). The random guessing result is shown as a line (geometric mean of the 21 errors) and a shaded area (span of the 21 errors).

The plot shows that all three exploratory-phase-free methods work drastically better than random guessing. With 30 model evaluations, these methods achieve an error that is smaller by a factor of 10^4^ compared to the mean error achieved by random guessing. The error plot of the three exploratory-phase-free methods and of the miracle-case essentially show the same performance: none of the exploratory-phase-free methods performs systematically worse than the miracle case. In other words, the parameter estimation comes as a byproduct and does not require additional model evaluations.

**Figure 4:**
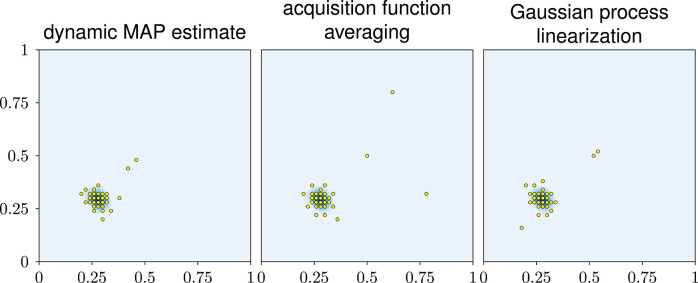
Visual impression of the designs in experiment 1. The blue background shows the true posterior of the input parameters. It consists of one mode close to (0.25, 0.25).

For a more intuitive perspective, Figure 4 shows the final designs of the three exploratory-phase-free methods. The posterior mostly consists of one mode. All three methods focus their design points almost entirely on this mode.

All three designs achieve an accurate likelihood estimate even in those parts of the parameter domain that are largely unexplored. This might seem surprising but it can be explained as follows: Recall that the likelihood estimator is defined as the average likelihood over the GP *U*, see Equation (2). This likelihood will only be large if, according to the GP *U*, it is reasonably plausible that the model output is close to the data. In this case, the likelihood estimates in the unexplored areas is small which, in turn, means that the GP, despite its uncertainty, is confident enough that the model output will not match the data in these areas.

#### 4.1.2. Computing Times

So far, we have only discussed the errors as a function of number of model evaluations. This makes sense if the model is computationally expensive. Still, each of the methods requires additional computing time for finding the optimal design points. This additional computing time is reported in Table 2.

**Table 2:** Number of sub-steps required for each approach and corresponding computing times (for 30 iterations).

	**# optimization of**	**# MCMC**	**# optimization of**	**Computing**
	**hyperparameters**		**acq. function**	**time [h:mm]**
Dyn. MAP	1	-	1	0:10
estimate				
Average	-	1	24	7:34
criterion				
Linearization	-	1	1	4:42

The first three columns show what computations are carried out in each iteration. The dynamic MAP estimate first optimizes the hyperparameters and then computes the acquisition function in each iteration. The linearization approach performs an MCMC and then computes the acquisition function. The average criterion approach first performs an MCMC and then computes the acquisition function for each realization of hyperparameters. In our case, we picked a small sample size of 24.

The last column shows the overall computing time required for finding the optimal design points (in 30 iterations). This time is to be added on top of the time required for the model evaluations. The table shows that a large part of the computing time is taken by the MCMC sampler. The dynamic MAP estimate does not require an MCMC and so is the fastest approach. However, these differences can be regarded as small when compared to the time required for evaluating the model function *u* (by assumption *u* is computationally expensive).

In summary, we recommend the use of dynamic MAP estimation for practical applications. While all three methods achieve roughly the same error level, dynamic MAP estimation is the fastest one and the easiest one to implement. In the following experiments, we will only consider the dynamic MAP estimation approach as the additional effort in the two other approaches does not provide any benefit.

#### 4.1.3. Sensitivity to the Prior

As explained in section 3.1.1, the approach of considering uncertain hyperparameters is only useful if finding a prior is easier in practice than finding the parameter values themselves. Furthermore, we introduced the hyperparameter prior with the intent that its influence diminishes as more and more model evaluations are done. In this section, we assess the sensitivity of the estimated solution of the inverse problem with respect to the choice of the hyperparameter prior.

To do so, we compare four different variants of the prior selected in the previous sections. The hyperparameter soft bounds for the four priors are shown in Table 1. The case “default” refers to the prior used before. The case “wide upper & lower” expands all bounds of the correlation length and the variance by a factor of 10. The case “wide upper” expands only the upper bounds of the correlation length and the variance. Finally, the case “narrow,” sets very narrow soft bounds for both correlation length and the variance, covering only one order of magnitude. The soft (95 %) upper bound for the correlation length with a value of 0.1 is deliberately chosen to not contain the value found by the map-method in the first experiment (which was approximately 0.34). To keep the results clear, we only apply the dynamic MAP estimation methods for all four priors.

**Figure 5:**
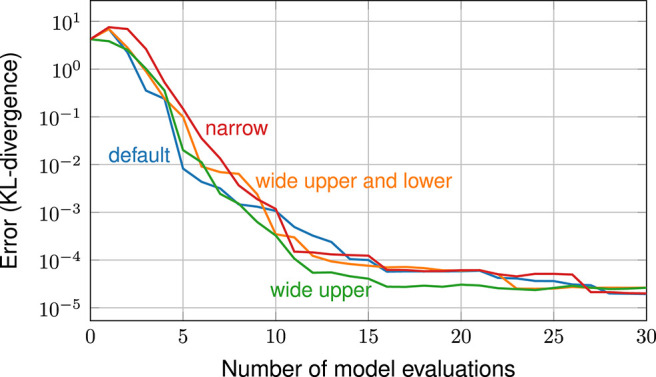
Error plot: comparison of different hyperparameter priors (with dynamic MAP estimation).

Error plots are shown in Figure 5. The four plots show similar error behavior and the differences are small compared to the advantage gained from considering uncertain hyperparameters instead of fixed ones. Overall, we conclude that the results are not overly sensitive to the choice of a prior. This means that, in practice, selecting a suitable hyperparameter prior is feasible and much easier than finding appropriate fixed hyperparameter values. This beneficial insensitivity is, among others, thanks to the chosen lognormal prior, as it does not exclude any values like a bounded distribution would do.

### 4.2. Experiment 2: Sorption

In the second experiment, we consider a slightly higher-dimensional problem with six input parameters. This time, the model function can be considered anisotropic, which means that each input parameter is expected to have a different influence on the model output, so we assign each input an individual correlation length. The estimation of individual correlation length parameters is called *automatic relevance determination* (Rasmussen and Williams, 2006; Shahriari et al., 2016).

This second experiment is based on simulations done by Nowak and Guthke (2016) who provided us with input-output data sets of their simulations. The current experiment is entirely done on this data set, so no additional simulation runs were carried out. Thus, the prior over the parameter domain is a discrete distribution defined by a sample of 51,000 points. The model output for each of these points is given in the data set as well, so a model evaluation here is simply a table lookup. Since we are measuring the computational effort in number of evaluations, this setup still works as an exemplary test case and the results are transferable to real cases, where the model function is an actual simulation.

The simulation behind the data set considers the transport of a contaminant through a low-conductivity clay layer in the subsurface. The important processes here are diffusion and sorption. Contaminant transport is modeled using the one-dimensional diffusion equation. Sorption is modeled using the Freundlich model (Nowak and Guthke, 2016; Fetter et al., 2018). The six input parameters consist of material parameters of the clay layer and the dissolved component (porosity, density, solubility and molecular diffusion), as well as two shape parameters within the Freundlich model. The output quantity of the model is a time series of the contaminant concentration at the lower boundary of the clay layer and consists of 20 concentration values. Measurement data is generated synthetically by picking one realization as the artificial truth and adding random noise. The precise setup of the simulation can be found in the original article by Nowak and Guthke (2016) but is not important for the study at hand.

In this experiment, we use GPs with squared exponential covariance. Furthermore, we use a random implementation of the sequential design: we pick a random subsample from the base sample of 51,000 points and use this subsample for computing the loss, see Equation (3), via MC estimate and for finding the minimum of the acquisition function via complete enumeration on this subsample. To get reliable results, we repeat each trial multiple times (random guessing: 21 times, all others 51 times) and consider the geometric mean errors.

**Figure 6:**
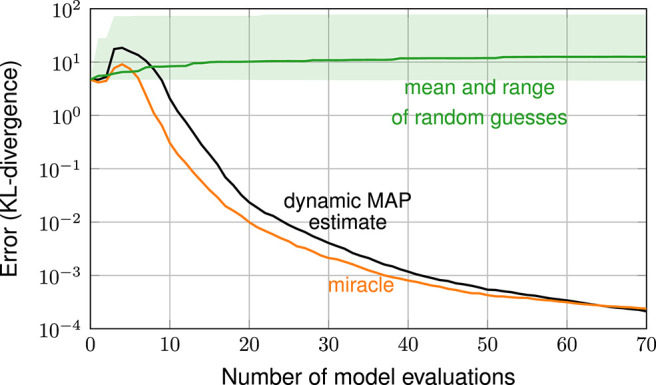
Error plot: exploratory-phase-free methods vs. random guessing.

Figure 6 compares the dynamic MAP estimation approach with random guessing and the miracle case. Errors are again averaged over multiple trials to provide a more robust error. We observe that picking hyperparameters at random is hopeless in this experiment. Among the 21 random trials, not even one was able to reduce the error at all. Our *dynamic MAP estimate* approach shows a performance comparable to *miracle*: at early times it lags behind the miracle case by about 4 iterations, later it catches up.

In summary, this section demonstrated that we can successfully find appropriate hyperparameters without an exploratory phase. Results of two more experiments are reported in the Supplementary Material. Next, we investigate, whether these findings allow us to eliminate the exploratory phase.

## 5. Can We Eliminate the Exploratory Phase?

After seeing that hyperparameter can successfully be estimated without an exploratory phase, we now assess, whether this means that we can eliminate the exploratory phase. To do so, we repeat the previous two experiments with exploratory phases of different sample sizes, and compare the performance. After that, we consider a new experiment (a variant of experiment 1), in which the solution is bi-modal. This experiment illustrates that the exploratory phase cannot always be eliminated. At the end of the section, we discuss how one might potentially identify whether or not the exploratory phase can be eliminated in a modeling task at hand.

### 5.1. Exploratory Phase in Experiments 1 and 2

For experiment 1, we employ exploratory phases with sample sizes 5, 10, 15 and 20. For the initial designs, we use latin hypercube sampling (LHS). Here, LHS designs serve as one representative of space-filling designs. Other flavors (e.g., maximin LHS) and other approaches (e.g., random sampling) are possible, too. Furthermore, we compare two different modes: first, with hyperparameters fixed after the exploratory phase and, second, with hyperparameters re-estimated via MAP estimate in every iteration.

**Figure 7:**
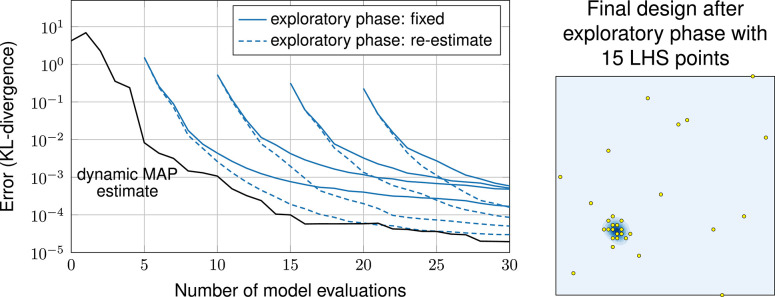
**Left**: Error plot, comparison of methods with and without exploratory phase. **Right**: Final design after exploratory phase with 15 latin hypercube samples.

The results are shown in Figure 7. Since the performance of the exploratory phase depends on the random initial sample, we repeated each design with 21 different initial designs. The plot shows the (geometric) mean error of these repetitions. The mean error looks smoother than the error of the other methods. This, however, is only an artifact of averaging and does not mean that an exploratory phase leads to a more robust estimate of the hyperparameters. In fact, each individual run of the 21 runs shows an error behavior that is as “unsmooth” as in the three exploratory-phase-free methods. Error bars are omitted here to avoid visual clutter. They are reported in the Supplementary Material.

The plot shows that re-estimating hyperparameters in each iteration works better than fixing them after the exploratory phase. In both cases, however, the exploratory phase leads to a larger error compared to the exploratory-phase-free methods. Only in one case, the exploratory phase led to a similar performance as the exploratory-phase-free methods—and that is the case with an initial sample of size 5 and with re-estimation, which is conceptually the closest one to exploratory-phase-free.

Note that Loeppky et al. (2009) suggest an initial sample of size of 20 for this case (10 times the dimension of the input). Following this suggestion here leads to a larger error compared to leaving out the exploratory phase altogether. From this we conclude that the context matters: while the suggestion by Loeppky et al. (2009) is useful in other uses of GPEs, it is actually computationally wasteful in the context of sequential design of computer experiments.

This experiment also shows that an exploratory-phase-free approach is only really beneficial, if the model function is computationally expensive and every model evaluation counts. As we see in Figure 7, the effect of the initial design wears off as the number of iterations increases.

**Figure 8:**
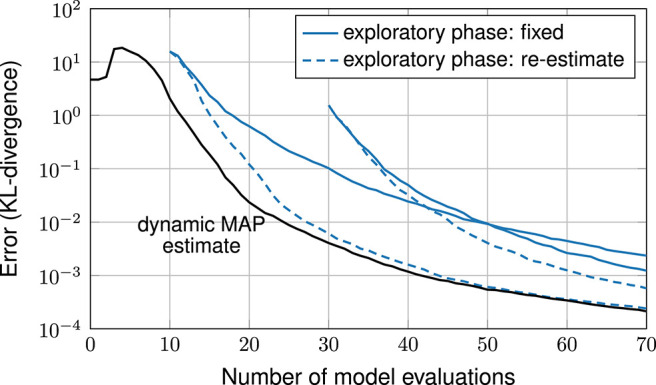
Error plot, comparison of methods with and without exploratory phase.

Next, in experiment 2, we employ exploratory phases with sample sizes 10 and 30. Here, initial samples are generated randomly, because the input parameters are statistically dependent (LHS designs are constructed for independent random variables). The results, shown in Figure 8, are similar to the one in the first experiments: with an exploratory phase, the performance is strictly worse compared to our exploratory-phase-free approach and smaller exploratory phases perform better than larger ones. Furthermore, the results confirm the common practice to re-estimate the hyperparameters being better than keeping them fixed.

### 5.2. Exploratory Phase in a Bi-Modal Problem

For this experiment, we solve the diffusion equation problem (Experiment 1) again, but use different locations for the temperature measurements. Instead of placing them on a 3 × 3-grid, we place nine measurement points equidistantly along the center line parallel to the x-axis. In this setup, we cannot distinguish between heat sources in the top and bottom half, so the resulting posterior will be symmetric with two modes.

**Figure 9:**
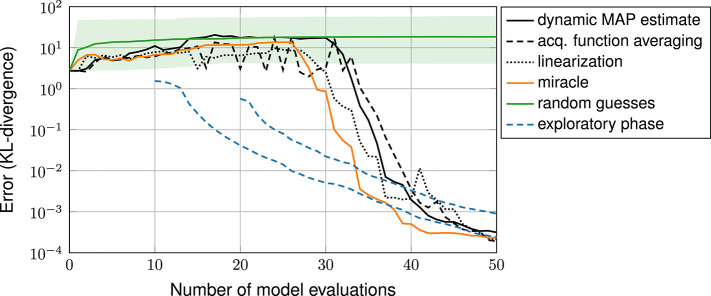
Bi-Modal problem: error plot, comparison of methods with and without exploratory phase.

Figure 9 shows error plots with and without exploratory phase. Here we see, that the exploratory-phase-free methods (including the miracle case) achieve a small error only after about 30 iterations. Yet, with an exploratory phase, the error decreases right away.

**Figure 10:**
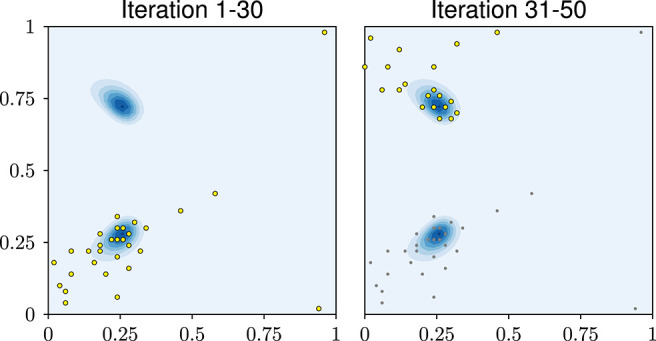
Bi-Modal problem: design points after 30 and 50 iterations.

To see why the exploratory-phase-free approach does not improve in accuracy in early iterations, Figure 10 shows the chosen design points. The first 30 design points all lie in the lower half of the domain, refining around one of the two modes. Only afterwards, the second mode is found and points are added around it as well. While this may seem inefficient, it is still a rational behavior: by construction, the GPE assumes the 18 output components to be statistically independent. When the first mode is found, it is very unlikely to the GPE that a second mode of similar accuracy exists, because this means that all 18 random variables were close to the data. In other words: this behavior is a rational consequence of the assumptions put into the GPE. As the experiment shows, an exploratory phase can solve this problem. Another solution might be to express a symmetry assumption in the GPE or to make the 18 output components statistically dependent.

This experiment highlights, that there are cases, where an exploratory phase can be beneficial for purposes other than hyperparameter estimation, so having an exploratory phase can be valuable.

### 5.3. Recommendation

Most examples in this work show a better performance if the exploratory phase is eliminated, while one example performs better with exploratory phase. This example, however, was specifically designed for this purpose. With these results, the authors suggest to eliminate the exploratory phase if (i) the model is computationally expensive and computational resources are limited—while surrogate models are already particularly beneficial in this context, the elimination can save even more time and resources— and (ii) the problem is expected to be unimodal. As stated above, this is often the case in hydro(geo)logy, where models tend to react monotonously to changes in parameters. But, of course, monotonicity is not a strict requirement. A problem can be unimodal without the model being monotonous.

## 6. Discussion and Conclusions

In this section, we discuss the generalizability of the results and provide a summary.

### 6.1. Generalizability to Other Problem Types

It remains an open question whether the results can be transferred to other problem types, such as Bayesian optimization or estimation of failure probabilities.

The authors conjecture that the results are transferable and that an exploration-phase-free estimation of hyperparameters is possible in other SDoE methods as well, but that the balance between exploration and refinement is important: exploration often creates larger distances between design points, while refinement creates smaller distances. By mixing large and small distances, the design points promise to be useful and informative for the estimation of hyperparameters. Again, if the acquisition function used in SDoE is designed to achieve a proper balance, then this is possible.

### 6.2. Summary

In this article, we investigated whether GP hyperparameters in sequential design of experiments methods can be estimated without a dedicated exploratory phase. To do so, the hyperparameters are re-estimated in each iteration, either by MCMC sampling from their marginal posterior or by maximum-a-posteriori (MAP) estimation. To make the acquisition function computationally tractable, three different simplified methods where presented: *dynamic MAP estimation, acquisition function averaging* and *Gaussian process linearization*. All three of these methods showed a performance similar to the so-called *miracle*-case, in which the optimal hyperparameters are used from the start. This means that these methods require only a few additional evaluation of the expensive-to-evaluate model function to find hyperparameters that are good enough for the task at hand (i.e., solving Bayesian inverse problems).

Furthermore, the numerical experiments show that the methods' performance is rather insensitive to the hyperparameter prior. This means that, in practice, selecting an appropriate prior is much easier than selecting the hyperparameters themselves. This article formulates a specific and practical rule for finding a hyperparameter prior and the numerical experiments confirm that this rule works well in practice.

From the three new methods, the dynamic MAP estimation is the easiest to implement and also the fastest, because it does not require an MCMC-sample from the hyperparameter posterior. Given that all three methods achieve a similar error, we can generally suggest the use of the dynamic MAP estimation method.

Further experiments showed that the exploratory phase can often be eliminated altogether. This could potentially simplify the overall procedure and make it faster. This, however, is not always the case. The limitations of eliminating the exploratory phase were shown in a specifically designed counter-example.

The research code for all of the results is available online^1^. Furthermore, the authors provide a python toolbox for the presented methodology (i.e., sequential design for Bayesian inverse problems, including hyperparameter estimation methods) with an easy-to-use interface. It is called *bali* and available on github as well^2^.

## Data Availability Statement

The datasets presented in this study can be found in online repositories. The data can be found here: https://github.com/MichaelSinsbeck/paper_hyperparameters-without-exploratory-phase.

## Author Contributions

MS: literature review, formulation of research question, coding and numerical experiments, and writing of manuscript. MH: additional literature review, scientific discussion, and rewriting of manuscript. WN: additional literature review, provisioning of test case 2, editing, and guidance. All authors contributed to the article and approved the submitted version.

## Conflict of Interest

The authors declare that the research was conducted in the absence of any commercial or financial relationships that could be construed as a potential conflict of interest.

## Footnotes

^1^github.com/MichaelSinsbeck/paper_hyperparameters-without-exploratory-phase

^2^github.com/MichaelSinsbeck/bali
