# Supplementary material for: Exploratory-Phase-Free Estimation of GP Hyperparameters in Sequential Design Methods—At the Example of Bayesian Inverse Problems
Source: Front Artif Intell. 2020 Aug 13;3:52. doi: 10.3389/frai.2020.00052 (PMC7861299; doi:10.3389/frai.2020.00052)
Supplement: Supplementary file 1 [file Data_Sheet_1.PDF]

Supplementary Material

1 MORE NUMERICAL EXAMPLES

Here, we present the results of two more numerical examples.

1.1 Experiment 3: Curve fitting

We consider a simple curve-fitting example, provided by Schöniger et al. (2014). A set of 15 (x,y) data points is given, where the x-values are spaced equidistantly between 0.25 and 4.75. We indent to fit a cosine function to these data points:

$$y = a \cdot \cos(bx + d) + d$$

The parameter vector (a, b, c, d) is uncertain with a multi-Gauss prior with mean $(2.6, 0.5, -2.8, 2.3)$ and covariance matrix

$$\begin{pmatrix} 0.46 & -0.07 & 0.24 & -0.14 \\ -0.07 & 0.04 & -0.05 & 0.02 \\ 0.24 & -0.05 & 0.30 & -0.16 \\ -0.14 & 0.02 & -0.16 & 0.30 \end{pmatrix}$$

For the measurement error (between cosine and data points), we assume a standard deviation of 0.6.

Figure S1 show the data points and the cosine obtained via least-squares optimization. Note that here, we are not interested in the least-squares solution, but in the posterior distribution of the model parameters (a, b, c, d).

Figure S2 shows the error plot, similar to the ones in the article. With dynamic MAP estimation, we achieve an average performance much better than by random guessing. After 30 iterations, the performance is close to the miracle case.

This plot also contains shaded areas indicating the range of errors after multiple runs of the methods. Notably, one of the runs with dynamic MAP estimation shows an upward spike at iteration 26. This means that in the worst case, not every iteration guarantees an improvement in error.

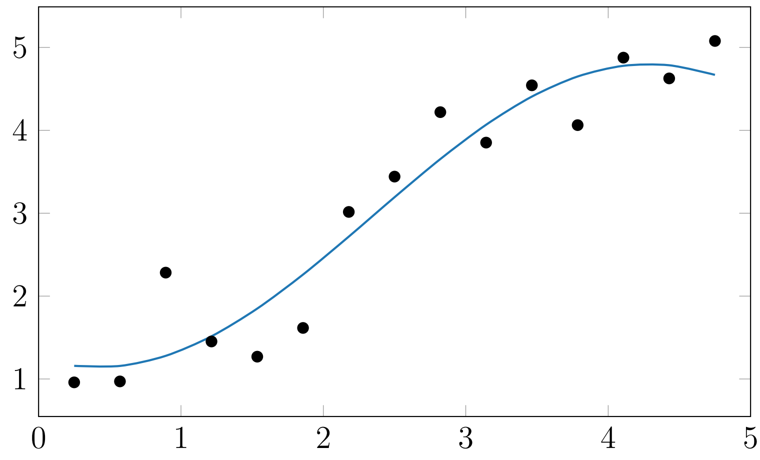

Figure S1. Data set and maximum least-squares models of experiment 3 (curve fitting).

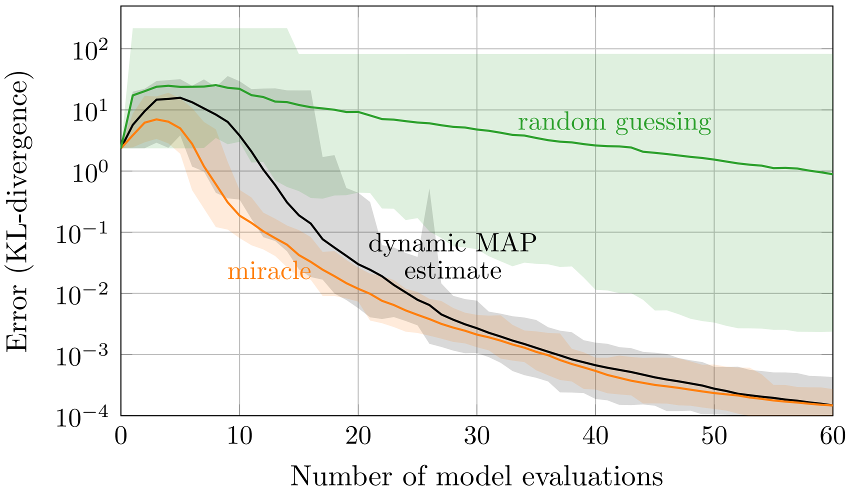

Figure S2. Error plot of experiment 3 (curve fitting). Shaded areas indicate the range of errors after multiple runs due to a stochastic implementation.

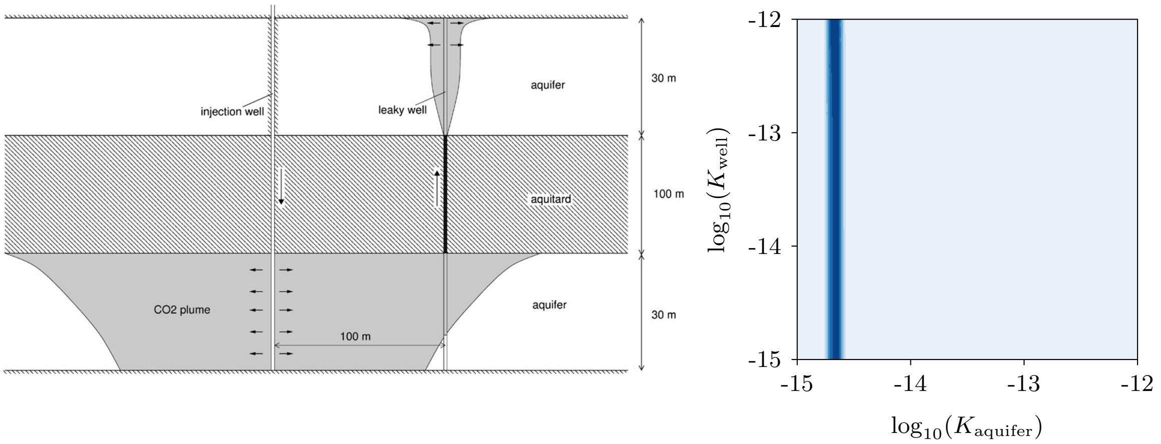

Figure S3. Left: model setup of experiment 4 (CO2 benchmark). Right: solution (posterior joint pdf) of experiment 4 (light blue = low density, dark blue = high density). The posterior is almost constant in y-direction.

1.2 Experiment 4: CO2 benchmark problem

The model in this example is a porous media flow simulation implemented in DuMu^x (Flemisch et al., 2011). It is a benchmark problem used and described by Oladyshkin et al. (2011b,a, 2013), so we provide only a brief summary of the model setup. The model is three-dimensional and considers two homogeneous aquifers that are separated by a non-conductive layer in between. The two layers are only connected by a leaky well that has been filled with a porous medium of different conductivity. At one position in the lower aquifer, the injection well, CO_2 is injected. At the injection point, a pressure measurement is taken every 10 days for 100 days. The two input parameters of the model are 1) the log-conductivity in the aquifers and 2) the log-conductivity in the well. A sketch of the setup is shown in Figure S3 on the left. Note that the figure only shows a two-dimensional slice of the three-dimensional setting.

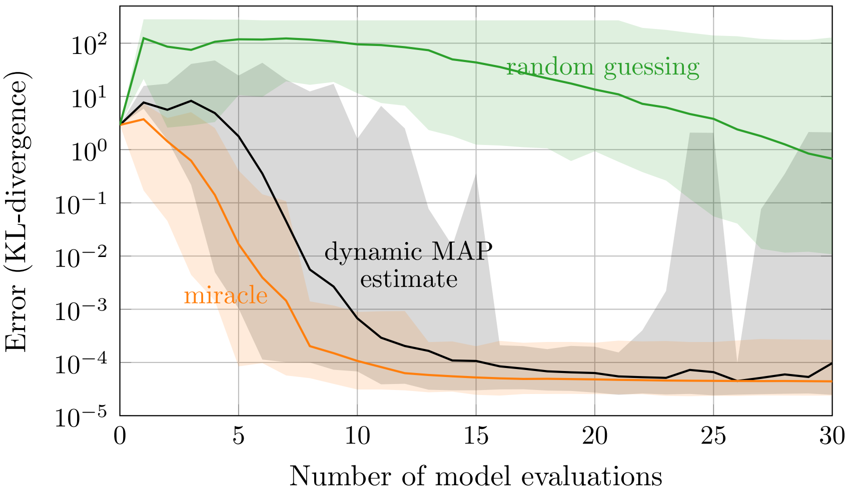

Figure S4. Error plot of experiment 4 (CO2 benchmark).

Data is created synthetically by running the model on a finer grid resolution, evaluating the pressure output and adding random noise. The finer grid resolution is necessary to avoid inverse crimes (Kaipio and Somersalo, 2007).

For both input parameters, we consider the base-10 logarithm of the conductivity and assume a uniform distribution for these between -15 and -12. Figure S3 on the right shows a color plot of the input parameter posterior distribution. The plot clearly shows that the posterior is almost insensitive to the second input parameter, which suggests that the model output is also insensitive to this parameter.

Figure S4 shows the error plot, similar to the ones in the article. Here, dynamic MAP estimation is, on average, three iterations behind the miracle case, and then stagnates at the same error level.

The gray shaded areas (the range of errors) show some severe upward spikes in later iterations (24-25 and 27-30). Since the average error is very small, the overall approach is still useful. However, it is of great interest for future research to reduce the variance in error to make the method more robust.

2 ERROR BARS

In this section, we show the error bars for plots that use a random implementation. As stated in the main article, each method was repeated multiple times with different random numbers. The error bars in the plots show the envelop of all repetitions (the area between minimum and maximum error).

Figure 6 in manuscript

Figure S5 shows the error bars of Figure 6 from the main manuscript. It shows that, at early times, the dynamic MAP estimate has a larger variance in the errors compared to the miracle case. At later times, after around 40 iterations, the dynamic MAP estimates reaches about the same variance in error compared to the miracle case. From there one, both methods show a very similar performance (both in average error and in error variance).

Figure 7 in manuscript

Figure S6 shows the error bars of Figure 7 from the main manuscript. When hyperparameters are re-estimated in each iteration, then the error has a spread of about one order of magnitude (left column). When

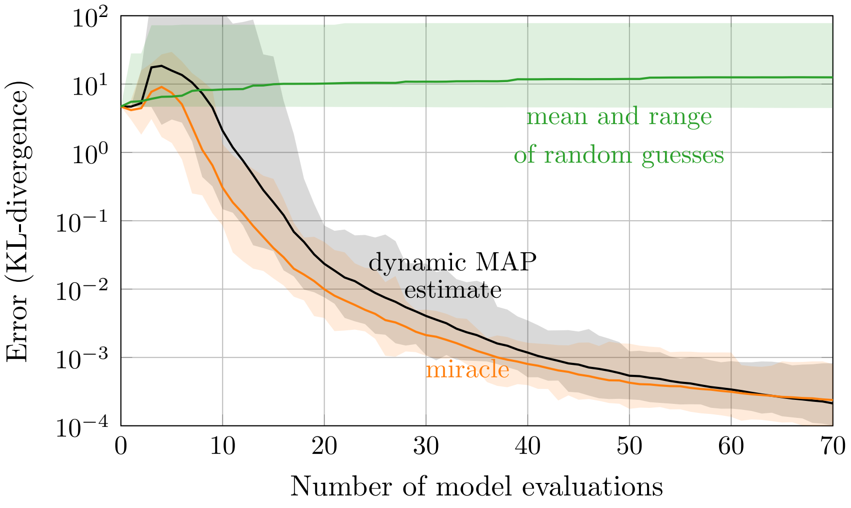

Figure S5. Error-bar version of Figure 6 from the main manuscript.

hyperparameters are fixed, then the spread is consistently larger (compared to re-estimation) and slightly increases over the number of iterations. Here, re-estimated hyperparamters achieve both a better and more consistent result (average errors are smaller and the spread is smaller).

Figure 8 in manuscript

Figure S7 shows the error bars of Figure 8 from the main manuscript. This figure confirms the findings from the previous figure. With hyperparameter re-estimation, results are more consistent and errors are smaller on average.

REFERENCES

Flemisch, B., Darcis, M., Erbertseder, K., Faigle, B., Lauser, A., Mosthaf, K., et al. (2011). DuMux: Dune for multi- {phase,component,scale,physics,...} flow and transport in porous media. *Advances in Water Resources* 34, 1102–1112

Kaipio, J. and Somersalo, E. (2007). Statistical inverse problems: Discretization, model reduction and inverse crimes. *Journal of Computational and Applied Mathematics* 198, 493–504

Oladyshkin, S., Class, H., Helmig, R., and Nowak, W. (2011a). A concept for data-driven uncertainty quantification and its application to carbon dioxide storage in geological formations. *Advances in Water Resources* 34, 1508–1518

Oladyshkin, S., Class, H., Helmig, R., and Nowak, W. (2011b). An integrative approach to robust design and probabilistic risk assessment for CO2 storage in geological formations. *Computational Geosciences* 15, 565–577

Oladyshkin, S., Class, H., and Nowak, W. (2013). Bayesian updating via bootstrap filtering combined with data-driven polynomial chaos expansions: methodology and application to history matching for carbon dioxide storage in geological formations. *Computational Geosciences* 17, 671–687

Schöniger, A., Wöhling, T., Samaniego, L., and Nowak, W. (2014). Model selection on solid ground: Rigorous comparison of nine ways to evaluate Bayesian model evidence. *Water Resources Research* 50, 9484–9513

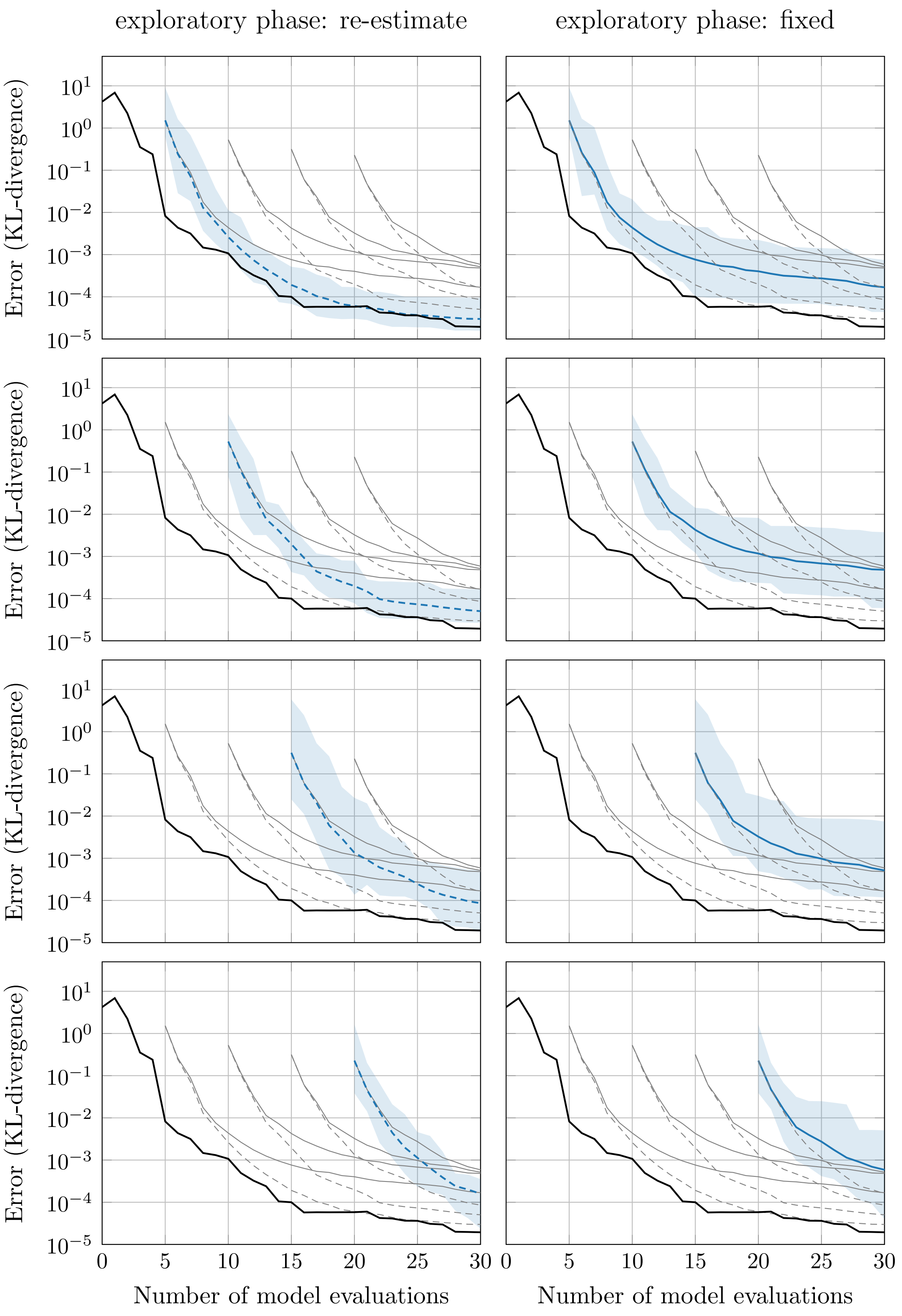

Figure S6. Error-bar version of Figure 7 from the main manuscript.

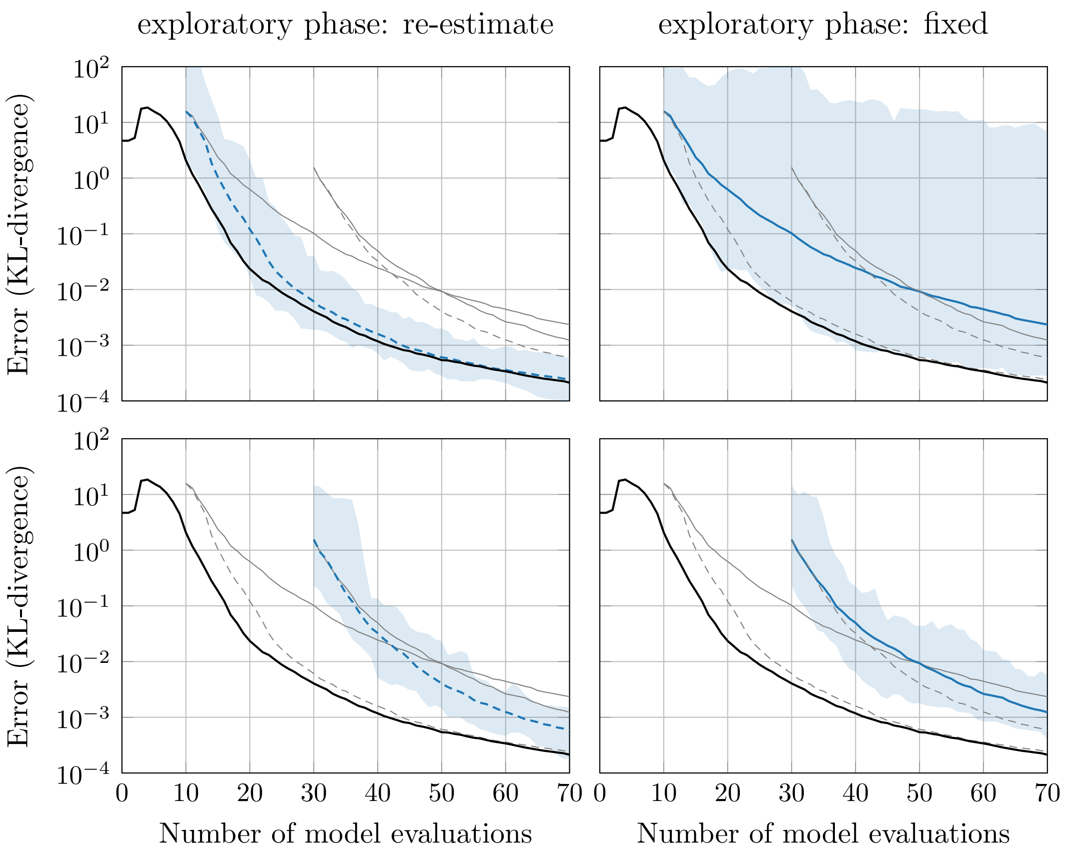

Figure S7. Error-bar version of Figure 8 from the main manuscript.
